# Executive Functions Rating Scale and Neurobiochemical Profile in HIV-Positive Individuals

**DOI:** 10.3389/fpsyg.2018.01238

**Published:** 2018-07-19

**Authors:** Vojislava Bugarski Ignjatovic, Jelena Mitrovic, Dusko Kozic, Jasmina Boban, Daniela Maric, Snezana Brkic

**Affiliations:** ^1^Department for Psychology, Faculty of Medicine, University of Novi Sad, Novi Sad, Serbia; ^2^Department for Psychology, Faculty of Philosophy, University of Novi Sad, Novi Sad, Serbia; ^3^Department for Radiology, Faculty of Medicine, University of Novi Sad, Novi Sad, Serbia; ^4^Department for Infectious Diseases, Faculty of Medicine, University of Novi Sad, Novi Sad, Serbia

**Keywords:** executive functions, anterior cingulate gyrus, behavior rating scale, HIV infection, MR spectroscopy

## Abstract

The set of complex cognitive processes, that are necessary for the cognitive control of behavior, known as executive functions (EF), are traditionally associated with the prefrontal cortex and commonly assessed with laboratory based tests and conventional neuroimaging. In an effort to produce a more complete and ecologically valid understanding of executive functioning, the rating scales have been developed in order to assess the behavioral aspects of EF within an everyday real-world context. The main objective of this study was to examine the relationship between behavioral aspects of EF measured by rating scale and neurometabolic profile in neurologically asymptomatic HIV-positive individuals under cART, measured using multi-voxel magnetic resonance spectroscopy (mvMRS). The sample comprised 39 HIV-positive adult male participants, stable on cART and 39 healthy HIV-negative volunteers. Both groups completed the Behavior Rating Inventory of Executive Function-Adult Version (BRIEF-A). HIV-positive group additionally underwent long-echo three-dimensional mvMRS to determine neurobiochemical profile in the anterior cingulate gyrus (ACG) of both hemispheres. Three dominant neurometabolites were detected: N-acetyl aspartate (NAA), the neuronal marker; choline (Cho), the marker of membrane metabolism and gliosis and creatine (Cr), the reference marker. Ratios of NAA/Cr and Cho/Cr were analyzed. The initially detected significant correlations between age, current CD4, BRIEF-A subscales Inhibit, Shift, Emotional Control, Plan/Organize, Self Monitoring and ratios of NAA/Cr and Cho/Cr in the dorsal and ventral part of the ACG, were lost after the introduction of Bonferroni corrections. Also, there were no significant differences between HIV–positive and HIV–negative group on any of BRIEF-A subscales. Such results possibly imply that stable cART regimen contributes to preservation of behavioral aspects of EF in asymptomatic HIV-positive individuals. Even though a subtle deficit in some aspects of EF might exist, it would not be manifest if behavioral aspect was assessed using EF rating scale. Further explanation might be that expected HIV-related changes in neurometabolic profile of the ACG under cART are not reflected in those behavioral aspects that are measurable by EF rating scale.

## Introduction

The executive functions (EF) are defined as the control or self-regulatory functions that organize and direct all cognitive activity, emotional response, and overt behavior (Miyake et al., 2000; Anderson, 2008; Duggan and Garcia-Barrera, 2015). The term “executive functions” represent an umbrella term for the interrelated functions that are responsible for purposeful, goal-directed, and problem-solving behavior in the everyday, “real world” environment (Goldstein et al., 2014). These functions are unique to our experience as human beings and critical for the successful and adaptive everyday functioning (Duggan, 2014). Given the central importance of the executive functions for the direction and control of the dynamic “real world” behavior, the reliance on traditional performance measurements can lead to a limited, incomplete assessment (Gioia and Isquith, 2004; Isquith et al., 2013). Resulting from an effort to produce a more complete and ecologically valid understanding of executive functioning, a number of rating scales have been developed in order to assess the behavioral aspects of executive function within an everyday real-world context; these scales can potentially serve as an ecological validity index for clinical or laboratory findings (Isquith et al., 2013; Duggan, 2014; Silver, 2014). In this sense, executive function rating scales were originally intended to serve as complementary measures to traditional assessment methods; however, studies have shown that performance-based and rating-based measurements assess different aspects of executive functioning and provide important complementary information to clinicians and researchers (Isquith et al., 2013; Toplak et al., 2013; Duggan, 2014; Silver, 2014).

According to Duggan (2014), some of the primary strengths of executive function rating scales are the ability to assess application of executive skills (rather than the functionality of their components), the capacity to capture executive characteristics of everyday functioning in clinical setting, the contributions of distinct information to executive function assessment, and the potential correlations with biological markers (Gioia et al., 2010; Isquith et al., 2013; Toplak et al., 2013). The presumed neurological basis for deficits in EF makes the relationship between markers of neuronal function and ratings on these scales an expected finding, especially in clinical populations (Isquith et al., 2013). Several studies reported associations between neural substrates and everyday executive functioning measured by rating scales (Isquith et al., 2013). The Behavior Rating Inventory of Executive Function (BRIEF; BRIEF-A) represents one of the most successful EF rating scales in daily activities, both in pediatric and adult clinical population. (Gioia and Isquith, 2004; Anderson et al., 2005; Wozniak et al., 2007; Garlinghouse et al., 2010; Kesler et al., 2011; Ghassabian et al., 2013; Hagen et al., 2016).

Frontal lobes are traditionally said to be involved in the executive functions. However, due to the high diversity of cognitive processes encompassed by the term “executive functions,” researchers put an effort to define functions within the executive domain according to the cognitive process that they are involved in. Functional neuroimaging studies have shed much light on this field, by identifying the cerebral regions engaged in the processing of certain cognitive task (Gazzaniga, 2004; MacPherson et al., 2015). However, these conclusions were not sufficient. The results of lesion studies also pointed to the frontal lobe regions as necessary for performing specific cognitive tasks (MacPherson et al., 2015).

The anterior cingulate gyrus (ACG) is a unique and important region in the brain circuitry, extensively connected to both the emotional and the cognitive regions. ACG lies in the medial part of both cerebral hemispheres, encompassing the corpus callosum. It is divided into two anatomically and functionally different regions, the ventral (or rostral) ACG (surrounding the genu of the corpus callosum) and the dorsal (or caudal) ACG (surrounding the body of the corpus callosum) (Palomero-Gallagher et al., 2009). Anatomic specificum of these regions (the whole dorsal and a half of ventral part) is the presence of Von Economo spindle neurons that are larger than pyramidal neurons, constructed for purposes of fast neurotransmission and high connectivity (Nimchinsky et al., 1999). The connections of these two parts differ greatly, as well as their proposed functions. The ventral part of ACG has connections with emotion (amygdala), autonomic (hypothalamus), memory (hippocampus) and reward-related (orbitofrontal cortex) regions of the brain. On the other hand, the dorsal part of ACG has rich connections with cognitive (lateral prefrontal) and motor-related (premotor and primary motor) regions, as well as with thalamic nuclei involved in pain- and motor-processing (Allman et al., 2001).

Functional roles of these two parts of ACG have been studied using functional neuroimaging studies and diffusion tensor imaging (DTI). DTI studies, that measured structural connectivity, largely confirmed patterns described in anatomical analyses (Stevens et al., 2011). Resting-state functional MR imaging (fMRI) revealed the connections between the ventral ACG and areas involved in affective processing, as well as between the dorsal ACG and sensorimotor and cognitive processing (Margulies et al., 2007). Task-related fMRI showed that motor-related tasks activated dorsal ACG, while simple emotions (sadness and happiness) activated more the ventral ACG. However, the sense of sadness contributed to the sense of pain, processed by the dorsal ACG. Cognitive control, conflict-monitoring, response-selection, error-detection and emotion-related appraisal are most likely processed also by the “cognitive” dorsal part of ACG. The ACG is thus the “affective” part, involved in processing emotion assessment, emotion-related learning and autonomic regulation (Etkin et al., 2011). Prior neurocognitive and neuroimaging studies in healthy population confirmed the association between anterior cingulate gyrus (ACG) and cognitive control functions, especially in conflict detection, performance monitoring and response selection (Botvinick, 2007; Alexander and Brown, 2017), as well as consolidation of memories (Nieuwenhuis and Takashima, 2011) and attention and reward-based learning (Hayden et al., 2011). Also, a functional division of ACG in ventral “affective” and dorsal “cognitive” subdivisions was confirmed (Mohanty et al., 2007).

Human Immunodeficiency Virus (HIV) infection has become a chronic condition after the introduction of combination antiretroviral therapy (cART), with the average lifespan of HIV-positive individuals reaching that of the HIV-negative population (Heaton et al., 2015). Currently, European AIDS Clinical Society guidelines strongly recommend the introduction of cART immediately after confirmation of HIV-seropositive status, regardless the immune status (Ryom et al., 2016). This early introduction of cART has significantly changed the overall picture of HIV-associated neurocognitive disorders, with a marked reduction in the prevalence of the most severe form, HIV-associated dementia (Chan and Brew, 2014) being up to 18% in the pre-cART era, and dropping down to 3–5% after the introduction of modern antiretroviral treatment (Heaton et al., 2011) The milder forms, asymptomatic neurocognitive impairment, and mild neurocognitive impairment remain highly prevalent in the cART era (Harezlak et al., 2011; Clifford and Ances, 2013). HIV-positive individuals have become an interesting clinical population for the assessment of cognitive status. Especially interesting are the EF, since they play a very important role in adherence to the antiretroviral therapy (Huerta et al., 2016). Recent studies showed impairments in multiple domains of executive functions in HIV-infected individuals (Heaton et al., 2011; Cattie et al., 2012; Giesbrecht et al., 2014), such as performance difficulties observed on cognitive shifting and complex sequencing tests (Marcotte et al., 1999; Mindt et al., 2003; Vazquez-Justo et al., 2003; Heaton et al., 2004;); response inhibition (Hinkin et al., 1999; Yadavalli, 2009); decision making (Hardy et al., 2006); abstract reasoning (Marcotte et al., 1999; Heaton et al., 2004); planning (Cattie et al., 2012) and working memory (Malagurski et al., 2017). In spite of peripherally successful cART application and the absence of a subjective experience, a certain degree of the cognitive deficit can still be observed (Malagurski et al., 2017). In addition, in the cART era, an important contribution to neuropsychiatric complications can be attributed to the antiretroviral drugs–nucleoside and non-nucleoside reverse transcriptase inhibitors in the first line (Treisman and Soudry, 2016).

One of the most commonly used advanced neuroimaging techniques for the detection of pathological process underlying neurocognitive impairment in HIV-positive population is magnetic resonance spectroscopy (MRS). MRS is a non-invasive diagnostic imaging technique used for detection of neurometabolite concentrations in small volumes of brain tissue. The pattern of changes in neurometabolic profile can characterize the type of ongoing pathological process in the brain parenchyma (Posse et al., 2013). Classical pattern of brain injury in chronic HIV infection consists of the decline in neuronal marker (*N*-acetyl-aspartate, NAA), and the increase in markers of membrane metabolism (choline-containing compounds, Cho) and glial proliferation (myoinositol, mI) (Ances and Hammoud, 2014). Recent MRS studies showed a reduction in neuronal marker in ACG in the HIV-positive subjects compared to healthy controls (Boban et al., 2017). Additionally, some differences were observed in the level of NAA in HIV-positive subjects with stimulant (alcohol, drugs) dependence, compared to both healthy controls and HIV-positive subjects without this dependence (Taylor et al., 2000). However, correlations between neurometabolite ratios and executive functions in chronic HIV infection have not been widely studied to date.

Although there are many recent studies that examined the use of EF traditional performance measurements in HIV-positive adult population, to date there are no studies that used EF rating scales. In addition, there are no studies that explored the relationship between neurometabolite ratios measured using advanced neuroimaging techniques such as multi-voxel MRS and behavior measured by EF rating scales, in HIV-positive adult population.

The first objective of this study was to assess the correlations between EF rating scale subtests and neurometabolite ratios obtained on the three-dimensional MRS in the ventral and dorsal ACG in chronically infected, virally suppressed HIV-positive individuals that are stable on long-standing cART.

The main hypothesis resulting from this objective was that there was a significant correlation between NAA/Cr, Cho/Cr ratios and EF rating scale subtests–better achievement on Working Memory, Emotional Control, Inhibit and Shift subtests would be followed by higher NAA/Cr and lower Cho/Cr ratios, especially in the “cognitive” dorsal part of the ACG.

Previous studies on neurocognitive status in HIV-positive individuals showed the presence of asymptomatic neurocognitive impairment even in subjects who are under stable cART regimen, thus supporting the assumption that HIV undoubtedly had certain negative influence on the central nervous system.

In well-controlled HIV infection, cognitive impairment is often subtle, discrete and commonly asymptomatic. Based on those findings, it can be assumed that the probability of detecting the cognitive deficit among people on a stable cART regimen on behavioral tests is relatively low, but possible.

The second objective of this study was to find out whether there was a difference between the group of HIV-positive individuals on stable cART and the group of HIV-negative healthy controls, on the EF rating scale subtests. It was hypothesized that there was a difference only on certain subtests, such as Working memory, Shift, Inhibit and Emotional control.

## Materials and methods

### Participants

This institutional ethical board-approved cross sectional study was conducted from 2015 to 2016 and comprised a total of 39 HIV-positive adult male participants, average age 42.18 years (range 25–66). All participants were Caucasians. All participants were chronically infected (over 1 year after diagnosed) and stable on cART (over 1 year on the same cART regimen). HIV-positive subjects were diagnosed with HIV infection using polymerase chain reaction (PCR) testing. After the initiation of cART, viral loads and current CD4 T-cell counts were closely monitored in each patient. Plasma viral load was under the detection threshold and current CD4 counts were over 250 cells/mm^3^ in all participants for at least 1 year. In addition, the regimen of cART remained unchanged for the same period of time. Based on the results of the screening neurocognitive test, International HIV Dementia Scale, (Sacktor et al., 2005), all participants were neurologically asymptomatic and able to work.

The criteria for inclusion of HIV-positive individuals in the study were: (1) age over 18, (2) normal conventional MR scan, (3) the presence of HIV infection (PCR verified), (4) HIV-seronegative status on PCR testing during previous 12 months (two negative PCR tests on a regular 6-months follow up), and (5) plasma viral load under the level of detection (<40 copies/mL). The exclusion criteria were the presence of focal or diffuse lesions in the brain (verified by conventional MR scan), active infiltrative or infective/opportunistic neurological disease, chronic neurological or psychiatric illness, comorbid disorders known to influence cognitive performance such as diabetes, cardiovascular diseases, hepatitis B or C, active abuse of alcohol and narcotic drugs. The route of infection was registered and in all HIV-positive participants and it was via sexual transmission. No illicit intravenous drug use or vertical transmission was detected in our cohort.

The control group consisted of 39 adult male participants, average age 42.21 years (range 25–65), Caucasian race, who were more than 18 years old and tested HIV-negative. The selection of the sample was performed randomly from general population. Healthy controls underwent neuropsychological testing with BRIEF-A. However, in these participants, neuroimaging was not performed due to the lack of clinical indication.

All subjects signed the fully informed written consent to participate in this study. Therefore, every participant was familiar with the research objectives. Demographic and clinical data of the participants in the study are summarized in Table 1 and descriptive statistics for the variables included in the study are summarized in Table 2. The difference between HIV+ and HIV– participants was not significant regarding educational achievement [*t*_(76)_ = −1.968, *p* > 0.05] as well as age [*t*_(76)_ = −0.010, *p* > 0.05].

**Table 1:** Clinical and sociodemographic characteristics for the study sample.

**Measure**		***N***	**Min**	**Max**	**Mean**	***SD***
Age	HIV+	39	25	66	42.18	11.37
	HIV–	39	25	65	42.21	11.18
Educational achievement (years)	HIV+	39	8	17	13.41	2.76
	HIV–	39	11	17	14.51	2.13
Duration of HIV infection (in months)	HIV+	39	17	234	75.26	61.79
Duration of cART (in months)	HIV+	39	17	234	67.95	57.71
Nadir CD4	HIV+	39	3.00	772.00	273.69	189.44
Current CD4	HIV+	39	210.00	2,000.00	617.33	364.37

**Table 2:** Descriptive statistics for the variables included in the study.

**Measure**		***N***	**Min**	**Max**	**Mean**	***SD***
BRIEF-A Inhibit	HIV+	39	8.00	20.00	13.03	3.15
	HIV–	39	8.00	19.00	12.41	3.14
BRIEF-A Shift	HIV+	39	6.00	14.00	9.23	2.18
	HIV–	39	6.00	17.00	8.92	2.62
BRIEF-A Emotional Control	HIV+	39	10.00	24.00	17.23	4.16
	HIV–	39	10.00	27.00	16.05	4.10
BRIEF-A Self Monitor	HIV+	39	6.00	14.00	9.59	2.38
	HIV–	39	6.00	15.00	8.82	2.06
BRIEF-A Initiate	HIV+	39	8.00	20.00	11.36	2.69
	HIV–	39	8.00	22.00	12.71	3.35
BRIEF-A Working Memory	HIV+	39	8.00	19.00	11.97	2.92
	HIV–	39	8.00	21.00	11.12	3.22
BRIEF-A Plan/Organize	HIV+	39	10.00	22.00	14.77	2.84
	HIV–	39	10.00	23.00	14.76	3.26
BRIEF-A Task Monitoring	HIV+	39	6.00	14.00	9.74	1.91
	HIV–	39	6.00	15.00	9.51	2.18
BRIEF-A Organization of Materials	HIV+	39	8.00	21.00	11.56	3.11
	HIV–	39	8.00	20.00	12.46	3.02
NAA/Cr - Ventral Part of the ACG right	HIV+	39	1.44	2.11	1.78	0.13
Cho/Cr - Ventral Part of the ACG right	HIV+	39	0.89	1.50	1.15	0.13
NAA/Cr - Ventral Part of the ACG left	HIV+	39	1.51	2.88	2.10	0.33
Cho/Cr - Ventral Part of the ACG left	HIV+	39	0.86	1.71	1.29	0.24
NAA/Cr -Dorsal Part of the ACG right	HIV+	39	1.18	2.49	1.71	0.26
Cho/Cr - Dorsal Part of the ACG right	HIV+	39	0.92	1.63	1.15	0.14
NAA/Cr - Dorsal Part of the ACG left	HIV+	39	1.07	2.31	1.69	0.26
Cho/Cr - Dorsal Part of the ACG left	HIV+	39	0.90	1.53	1.15	0.14

### Measures

Behavior Rating Inventory of Executive Function-Adult Version–BRIEF-A (Roth et al., 2005) is the standardized rating scale developed to assess behavior manifestations of executive functions in adults, aged 18–90 years. The BRIEF-A consists of Self-Report and Informant Report Forms. The Self-Report Form, used in this study, provides an understanding of the individual's perspective with respect to their own difficulties in self-regulation. The questionnaire contains 75 items within nine non-overlapping clinical scales: Inhibit, Shift, Emotional control, Initiate, Working Memory, Plan/Organize, Organization of Materials, Task Monitor and Self-Monitor. Responses were indicated on a 3-point scale labeled never, sometimes, often, where lower score represents better performance. Reliability analyses, measured by Chronbach's Alpha, revealed high internal consistency of all clinical scales for this study sample (Inhibit α = 0.808, Shift α = 0.701, Emotional Control α = 0.878, Self Monitor α = 0.811, Initiate α = 0.725, Working Memory α = 0.778, Plan/Organize α = 0.670, Task Monitor α = 0.651, Organization of Materials α = 0.813).

### Multi-voxel magnetic resonance spectroscopy protocol

**Figure 1:**
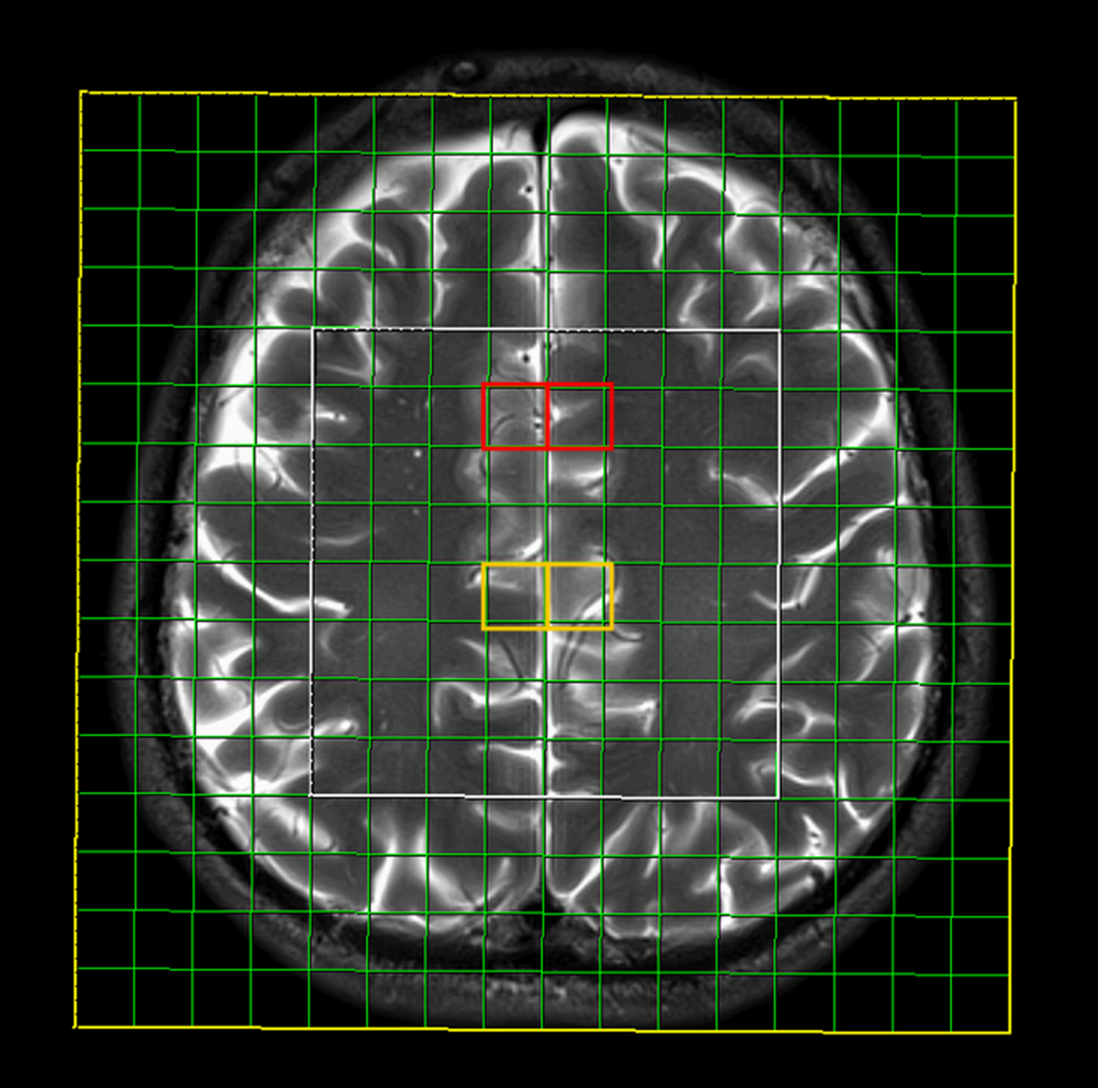
Multivoxel network with selected locations in the ventral (red squares) and the dorsal (yellow squares) parts of the anterior cingular gyrus, where MR spectra were analyzed.

Magnetic resonance spectroscopy was performed immediately after neuropsychological testing on a 3T MR scanner (Siemens Trio Tim, Erlangen, Germany), using an 8-channel head array coil. Conventional MR scan consisted of multisequentional and multiplanar tomograms, necessary for excluding focal or diffuse white and gray matter lesions, and for localization of multivoxel network. Conventional MR imaging consisted of: T1-weighted sagittal, T2-weighted and FLuid Attenuation Inversion Recovery (FLAIR) axial and T2-weighted coronal tomograms, as well as thin-sliced 3D T1-weighted magnetization prepared rapid acquisition gradient echo (MPRAGE) sagittal tomograms. Three-dimensional multi-voxel magnetic resonance spectroscopy was performed using point-resolved spectroscopy with a long echo time (time of repetition 1,700 ms/time of echo 135 ms). The volume of interest (VOI) was 80 × 80 × 80 mm, with a slice thickness of 10 mm, positioned parallel to the axial images. Total scan time was 7:17 min (weighted phase-encoding scheme was applied). Saturation planes (for saturating signals of surrounding tissues) were manually positioned along the margin of the VOI. The automatic, volume selective shimming method was used to optimize homogeneity of the magnetic field. VOI was positioned in the same way in every subject in order to achieve the reproducibility, while analyzed voxel positions were chosen manually. Multivoxel network was placed in the supracallosal white matter and gray matter, comprising gray matter of anterior and posterior cingulate gyrus and white matter of frontal centrum semiovale (Figure 1). Due to the reasons stated in Introduction, four localizations were chosen out of possible 64 voxels (8 × 8 network): two placed in the ventral part of anterior cingulate gyrus (one in the left and one in the right hemisphere) and two placed in the dorsal part of anterior cingulate gyrus (also one in the left and one in the right hemisphere). The spectra were imported to a digital workstation, where a dedicated manufacturer's software for spectroscopy was applied for baseline corrections, peak identification and calculation of the ratios throughout analyzed voxels. Spectra obtained on short echo time MRS were analyzed by identifying peaks of N-acetyl aspartate (NAA) at 2.02 ppm, choline (Cho) at 3.2 ppm, and creatine (Cr) at 3.0 ppm. Ratios of NAA/Cr and Cho/Cr were calculated for each voxel. Spectra from left and right side were analyzed separately, due to possible lateralization in some executive functions (emotional control and working memory, for example).

### Analysis

#### Variables

Variables in this research were obtained as scores from nine BRIEF-A scales (constructed by adding the items belonging to a certain scale in a sum score) as well as ratios of NAA/Cr and Cho/Cr in dorsal and ventral part of the anterior cingulate gyrus (ACG) on both sides, in order to determine the relationship between executive functions and neurobiochemical profile. Due to the presumed lateralization of some executive functions (although chronic HIV-related changes affect the brain volume diffusely), we analyzed each voxel separately. Age, educational achievement, duration of HIV infection, duration of cART therapy, nadir CD4 count (the lowest CD4 count in patient's history), and current CD4 counts were examined in relationship with executive functions. Also, these parameters were examined as potential control variables in a regression model.

#### Data analyses

All statistical analyses were performed using IBM SPSS software (Version 23.0, Chicago, IL, USA). Pearson's correlation analyses were conducted to estimate the relationship between neurobiochemical profile in normal-appearing brain tissue and executive functions, clinical parameters, sociodemographic variables and executive functions as well as the relationship between clinical parameters, sociodemographic variables, and neurobiochemical profile. Four Pearson's correlation analyses were conducted separately regarding specific areas of interest. In order to investigate the difference on nine BRIEF-A scales between HIV+ and HIV– individuals, nine *t*-tests were conducted. Moreover, five hierarchical regression analyses were conducted to estimate the unique contributions of neurobiochemical changes in normal-appearing brain tissue, in the prediction of executive functions.

Due to the small study sample and many hypothesis tested, the Bonferroni correction was introduced in order to control family wise error rate (FWE) and avoid making Type I error (Armstrong, 2014). The Bonferroni correction was used for Pearson's correlation tests since multiple comparisons were tested in a way that the alpha value (*p*-value) was adjusted by the number of comparisons being performed. To perform the Bonferroni correction, the *p*-value (*p* = 0.05) was divided by the number of comparisons for each test separately. For the first Pearson's analysis where the goal was to determine the relationship between clinical parameters, sociodemographic variables, and executive functions in HIV+ individuals, the *p*-value was calculated and the significance value was set at *p* = 0.0009. For the second Pearson's correlation analysis where the goal was to investigate the relationship between EF and neurobiochemical profile in HIV-positive individuals, the significance value was set at *p* = 0.003. In order to investigate the relationship between clinical parameters, sociodemographic variables, and neurobiochemical profile in HIV-positive individuals, statistical significance was set at *p* = 0.001. Finally, in order to determine the relationship between executive functions and sociodemographic variables in HIV-individuals, the significance value was set at *p* = 0.003.

However, since this correction was too rigorous and all statistically significant results were lost, we presented and discussed the obtained results as if the Bonferroni correction was not applied.

## Results

In order to determine the relationship between sociodemographic variables, clinical parameters and executive functions, Pearson's correlation test was performed (Table 3). Statistically significant positive correlations were obtained between the variable Age and BRIEF-A scale Shift as well as between current CD4 count and BRIEF-A scale Working Memory.

**Table 3:** Correlations between sociodemographic variables, clinical parameters and BRIEF-A measures in HIV-positive individuals.

	**Age**	**Educational achievement (years)**	**HIV (months)**	**cART (months)**	**Nadir CD4**	**Current CD4**
Inhibit	0.019	−0.104	0.011	0.001	0.274	0.229
Shift	0.391^**^	−0.116	0.249	0.260	0.026	0.216
Emotional control	0.224	−0.276	0.206	0.220	−0.116	0.067
Self monitoring	0.112	−0.062	−0.079	−0.043	0.107	0.039
Initiate	0.074	0.040	0.002	−0.029	−0.058	0.174
Working memory	0.059	−0.002	0.048	0.026	0.146	0.324^*^
Plan/Organize	0.210	−0.188	0.109	0.095	0.056	0.290
Task monitoring	0.134	−0.059	0.125	0.141	0.020	0.171
Organization of materials	−0.044	−0.122	0.022	0.001	−0.057	0.060

** *p < 0.01*,

* *p < 0.05*.

Pearson's correlation analysis was performed in order to determine the relationship between sociodemographic variables and EF (Table 4). Significant positive correlation was obtained between the educational achievement and Working Memory.

**Table 4:** Correlations between sociodemographic variables and BRIEF-A measures in HIV-negative individuals.

	**Age**	**Educational achievement (years)**
Inhibit	−0.178	0.157
Shift	0.175	0.115
Emotional control	−0.001	0.012
Self monitoring	0.205	0.159
Initiate	0.004	0.105
Working memory	0.108	0.319^*^
Plan/Organize	0.225	0.017
Task monitoring	−0.014	0.274
Organization of materials	0.236	0.101

* *p < 0.05*.

Pearson's correlation analysis was performed in order to determine the relationship between executive functions and neurobiochemical profile (Table 5). Statistically significant negative correlations were obtained between NAA/Cr at the dorsal part of the ACG (right) and Inhibit, Shift, Emotional Control, Plan/Organize as well as between Cho/Cr at the dorsal part of the ACG (right) and Self Monitoring. Furthermore, statistically significant negative correlations were obtained between NAA/Cr at the dorsal part of the ACG (left) and Shift, Emotional Control, and Self Monitoring.

**Table 5:** Correlations between neurobiochemical profile and executive functions in HIV-positive individuals.

	**1**	**2**	**3**	**4**	**5**	**6**	**7**	**8**
1 NAA/Cr - VP ACG right	1							
2 Cho/Cr - VP ACG right	−0.238	1						
3 NAA/Cr - VP ACG left	−0.036	−0.199	1					
4 Cho/Cr - VP ACG left	0.097	0.009	−0.026	1				
5 NAA/Cr - DP ACG right	−0.180	0.105	0.038	−0.134	1			
6 Cho/Cr - DP ACG right	−0.449^**^	0.497^***^	0.066	−0.051	0.302	1		
7 NAA/Cr - DP ACG left	−0.242	0.126	−0.074	−0.106	0.813^***^	0.183	1	
8 Cho/Cr - DP ACG left	−0.397^**^	0.443^***^	−0.042	0.000	0.094	0.658^***^	0.381^**^	1
Inhibit	−0.054	0.005	−0.267	0.247	−0.347^*^	0.003	−0.246	0.103
Shift	−0.004	−0.004	0.150	0.135	−0.399^**^	−0.081	−0.385^**^	0.013
Emotional control	0.005	0.084	−0.153	0.078	−0.397^**^	0.023	−0.388^**^	0.058
Self monitoring	0.119	−0.220	−0.159	0.114	−0.370^*^	−0.326^*^	−0.333^*^	−0.164
Initiate	−0.115	−0.009	−0.069	0.043	−0.118	0.153	−0.147	0.105
Working memory	−0.027	0.026	0.057	0.131	−0.100	0.050	−0.088	0.087
Plan/organize	−0.078	−0.069	−0.096	0.191	−0.364^*^	−0.127	−0.283	−0.016
Task monitoring	−0.248	−0.067	−0.072	0.155	−0.183	0.015	−0.218	0.048
Organization of materials	−0.217	−0.052	−0.121	0.065	−0.091	−0.056	−0.026	0.085

*VP ACG, ventral part of the anterior cingulate gyrus; DP ACG, dorsal part of the anterior cingulate gyrus*.

*** *p < 0.001*,

** *p < 0.01*,

* *p < 0.05*.

In order to determine the relationship between sociodemographic variables, clinical parameters and neurobiochemical profile in HIV-positive individuals, Pearson's correlation test was performed. The results are summarized in Table 6: statistically significant correlations were obtained between NAA/Cr at the ventral part of the ACG (left) and nadir CD4 count, NAA/Cr at the dorsal part of the ACG (right)and age, as well as between the duration of cART, NAA/Cr at the dorsal ACG (left) and age.

**Table 6:** Correlation between sociodemographic variables, clinical parameters and neurobiochemical profile in HIV-positive individuals.

	**NAA/Cr VP ACG right**	**Cho/Cr VP ACG right**	**NAA/Cr VP ACG left**	**Cho/Cr VP ACG left**	**NAA/Cr DP ACG right**	**Cho/Cr DP ACG right**	**NAA/Cr DP ACG left**	**Cho/Cr DP ACG left**
Age	0.04	0.235	0.174	0.115	−0.530^***^	0.074	−0.478^***^	0.152
Educational achievement (years)	−0.061	0.014	−0.069	−0.064	0.243	0.202	0.171	0.096
HIV (months)	0.001	0.082	0.034	0.192	−0.272	0.134	−0.241	0.19
cART (months)	−0.007	0.08	0.092	0.178	−0.309^*^	0.136	−0.291	0.183
Current CD4	−0.077	0.162	−0.025	0.049	−0.010	0.019	0.014	0.147
Nadir CD4	−0.084	0.184	−0.314^*^	0.183	−0.002	0.037	0.056	0.138

*VP ACG, ventral part of the anterior cingulate gyrus; DP ACG, dorsal part of the anterior cingulate gyrus*.

*** p < 0.001, and

* *p < 0.05*.

Nine *t*-tests were conducted in order to investigate the difference between HIV-positive and HIV-negative individuals on nine BRIEF-A scales. Observing the results (Table 7), we could state that there is no difference between age-matched HIV-positive and HIV-negative individuals with regard to EF.

**Table 7:** *T*-tests results comparing HIV-positive and HIV-negative individuals on executive functions.

	**Levene's**		***T*****-test**						
								**95% Confidence interval of the difference**	
**BRIEF-A**	***F***	***p***	***t***	***df***	***p***	***MD***	***SED***	***Lower***	***Upper***
Inhibit	0.242	0.624	0.877	76	0.383	0.615	0.702	−0.782	2.013
Shift	0.005	0.945	0.562	76	0.576	0.307	0.547	−0.782	1.397
Emotional Control	0.295	0.589	1.261	76	0.211	1.179	0.935	−0.683	3.042
Self Monitoring	2.736	0.102	1.525	76	0.131	0.769	0.504	−0.235	1.774
Initiate	1.830	0.180	−1.973	76	0.052	−1.358	0.688	−2.730	0.012
Working Memory	0.701	0.405	1.215	76	0.228	0.846	0.696	−0.541	2.233
Plan/Organize	0.415	0.521	0.000	76	1.000	0.000	0.693	−1.380	1.380
Task Monitoring	0.376	0.542	0.496	76	0.622	0.230	0.465	−0.696	1.157
Organization of Materials	0.009	0.926	−1.292	76	0.200	−0.897	0.694	−2.281	0.486

*MD, mean difference; SED, std. error difference*.

Hierarchical regression analyses were performed in order to determine the influence of the NAA/Cr and Cho/Cr ratios at the dorsal ACG (left and right) on following BRIEF-A scales: Inhibit, Shift, Emotional Control, Self-monitoring, and Plan/Organize. Age was used as the control variable in the first step, the current CD4 count in the second step, and the NAA/Cr and Cho/Cr ratios at the dorsal ACG (left and right) in the third step. The results (Tables 8–17 in Supplementary Materials) showed that age had a significant influence on clinical scale Shift. However, none of the metabolite ratios had significant influence on the achievement BRIEF-A scales.

## Discussion

Recent studies showed that HIV-positive individuals have a significantly extended lifespan due to the introduction of cART (Heaton et al., 2015). However, this resulted in a more frequent occurrence of asymptomatic neurocognitive impairment represented with subtle deficit in multiple domains of EF (Heaton et al., 2011; Cattie et al., 2012; Giesbrecht et al., 2014). Majority of studies that reported deficits in HIV-positive subjects, assessed the EF using limited, laboratory-based tests, known as traditional performance measures. Over time, however, clinicians and researchers revealed that these tests could not always detect impairment in patients who had clear executive dysfunction in their everyday lives, while, at the same time, in patients with no evidence of executive problems outside of the test setting, tests indicated impairment (Pennington and Ozonoff, 1996). Due to aforementioned reasons, the standardized rating scales of real world behavior are becoming increasingly used as a supplement to the traditional laboratory tests (Silver, 2014).

To date, to the best of our knowledge, there are no HIV-related studies which used rating scales in the assessment of EF. This was the main reason for the authors of this study to use the behavior rating scale of EF in chronically infected, virally suppressed HIV-positive subjects under long-standing cART and in correspondent group of HIV-negative individuals, in order to detect potential differences on nine subscales of BRIEF-A between these two groups. The results showed that there were no significant differences between HIV-positive and HIV-negative individuals on the subscales Inhibit, Shift, Emotional control, Initiate, Working Memory, Plan/Organize, Organization of Materials, Task Monitor and Self-Monitor. This was a rather surprising result, since at least subtle differences were expected primarily on Working memory, Shift, Inhibit and Emotional control subscales, that represented basic/core cognitive processes of EF and played an important role in organizing behavior. Also, these specific aspects of EF have been most thoroughly studied in clinical HIV-positive populations.

Some subtle, though asymptomatic neurocognitive deficits can be observed in HIV-positive individuals under cART using traditional performance measure tests (Malagurski et al., 2017). However, in our study, no significant deficit was recorded in any of nine scales of EF. Potential explanation could be that, even if a certain degree of disturbance existed in tested components of EF, this deficit was not pronounced enough to be observed in the behavioral domain. This could imply that in HIV-positive individuals under stable cART, who are functional in everyday activities and capable to work, there are no prominent deficits in the behavioral domain, even though they can be detected on traditional performance measure tests. Furthermore, it might be true that a behavioral deficit has to be more prominent to cause the disturbances in everyday, real life situations in HIV-positive patients. Indirect confirmation for this conclusion was that our sample included HIV-positive who were highly functional in everyday activities and that the level of compliance was high (reflected in plasma viral load under the detection limit). Additional explanation could lie in potential functional neuronal reorganization and brain plasticity that prevents further deprivation of executive functions. A recent study by Sanford et al. recently raised the issue whether the functional remodeling in the pathways involved in cognition existed in HIV-positive individuals under cART. In a recently published 2-year follow-up study, a better interval performance was observed on several neurocognitive tasks, explained as the result of a beneficial effect of the on-time introduction of cART and long-standing stable aviremia (Sanford et al., 2018).

The second main objective of the study was to examine the presence of correlations between EF rating scale subtests and neurometabolite ratios in the dorsal and ventral part of the ACG, obtained using multi-voxel MRS in chronically infected, virally suppressed HIV-positive individuals under stable and long-standing cART. The idea to explore these correlations came from everyday clinical practice, since we felt that obtained results could contribute to the better clinical management of HIV-positive individuals. Additionally, if significant correlations between neurometabolite ratios and executive functions were observed, it would be the additional confirmation of the key neuroanatomic role of ACG in EF, especially given that these correlations have never been examined in HIV-positive subjects. Also, the data obtained on this easy and fast assessment scale, suggesting the presence of neurodegeneration in the domains related with everyday activities, could lead the clinical decision on introducing further neuroimaging and detailed neurocognitive studies. That way, new protocols for the assessment of neurocognitive status in an individual patient could be established, based on the speed, efficacy and cost-benefit analysis, thus contributing to the better initial triage of the patients eligible for the cognitive assessment.

We decided to examine correlations between BRIEF-A subscales and neurometabolite ratios in the ventral and dorsal parts of the ACG using Pearson's correlation analysis. Statistically significant correlations were obtained between NAA/Cr at the dorsal part of the ACG (right) and Inhibit, Shift, Emotional Control, Plan/Organize, as well as between Cho/Cr at the dorsal part of the ACG (right) and Self Monitoring. Furthermore, statistically significant correlations were obtained between NAA/Cr at the dorsal part of the ACG (left) and Shift, Emotional Control, and Self-Monitoring.

With regard to the obtained correlations, one can see that the majority of significant correlations involved scales of Behavioral regulation domain: Inhibit, Shift, Emotional Control and Self-Monitoring. Achievement on all these scales reflects the ability to maintain appropriate regulatory control of one's own behavior and emotional responses. This includes appropriate inhibition of thoughts and actions, flexibility in shifting problem-solving set, modulation of emotional response, and monitoring one's actions. Appropriate behavioral regulation is likely to be a precursor of appropriate meta-cognitive problem solving that successfully guides active and systematic problem solving, and more generally supports appropriate self-regulation (Isquith et al., 2013). Lower concentrations of neuronal marker (NAA/Cr) in the dorsal ACG (left and right) are connected to the poorer achievement on these scales. This result clearly confirmed that the dorsal ACG is the key structure in behavioral regulation, problem solving and monitoring of thoughts and actions. The decrease of NAA/Cr in the left and right dorsal ACG is related to the deprivation of inhibitory control and impulsivity, leading to the decline of the ability to resist impulses and the ability to stop one's own behavior at the appropriate time. The ability to move with ease from one situation, activity, or aspect of a problem to another as the circumstances demand is also deprived. Key aspects of shifting include the ability to make transitions, tolerate change, problem-solve flexibly, switch or alternate attention and change focus from one mindset or topic to another, implying that the progressive decline of all these shifting aspects occurs (Isquith et al., 2013). The decrease of NAA/Cr ratios in the dorsal ACG (left and right) was correlated with poor achievement on Emotional Control subscale, primarily with regard to the impact of EF problems on emotional expression and assesses an individual's ability to modulate or control his or her emotional responses. It is interesting that Self-Monitor scale was the only scale that showed correlations with Cho/Cr ratios in the right ACG. The decrease in both Cho/Cr and NAA/Cr ratios in the dorsal ACG (left and right) is correlated with impairment in the aspects of social or interpersonal awareness, since this scale captures the degree to which an individual perceives himself as aware of the effect that his/her behavior has on others (Isquith et al., 2013). From other scales involved in Metacognition domain, only the Plan/Organize scale showed positive correlation with NAA/Cr in the dorsal ACG on the right. The Plan/Organize scale measures an individual's ability to manage current and future-oriented task demands. This scale consists of two components: plan and organize. The Plan component captures the ability to anticipate future events, to set goals, and to develop appropriate sequential steps ahead of time in order to carry out a task or activity. The Organize component refers to the ability to bring order to information and to appreciate main ideas or key concepts when learning or communicating information (Isquith et al., 2013). Obtained correlations imply the key role of the dorsal ACG in the relationship between behavioral changes in everyday setting with the decrease in neuronal markers in chronic HIV-positive subjects.

The authors wanted to satisfy more strict statistical criteria and, therefore, the Bonferroni corrections were performed. However, after the introduction of the Bonferroni corrections, none of the initially detected correlations remained significant. The authors expected that correlations between those variables should exist, based on prior studies, both using multi-voxel MRS and other advanced neuroimaging techniques. Therefore, we decided to discuss the potential explanations for the absence of significant correlations after the introduction of this strict statistical criterion.

Given that a decrease in some neurometabolite concentrations in chronically infected virally suppressed HIV-positive subjects was observed in a prior study (Boban et al., 2017), we expected that this decrease could be correlated with disturbances in some behavioral aspects that are coordinated with EF. The lack of expected correlations after the introduction of Bonferroni corrections can be explained with several arguments. The first argument was that our sample was consisted of neuroasymptomatic HIV-positive subjects, under stable cART regimen, functional in everyday activities and able to work. We speculated that, although the presence of HIV can affect the neurometabolite ratios, the good clinical control of the disease prevented the overt profile in behavioral aspects (that could be correlated with expected deficits in executive functions). The lack of differences between HIV-positive and control subjects on BRIEF-A subscales indirectly confirmed that behavioral aspects of executive functions in asymptomatic and working HIV-positive individuals are not inferior to those in healthy controls. An additional explanation could follow the direction of potential neuronal remodeling and brain plasticity that prevented manifest deprivation of executive functions. The other argument deals with the issue of successful use of self-assessment scales (such as BRIFEF-A) in behavioral analyses of HIV-positive subjects, since the participants could have given socially appealing answers in order to present themselves as functional. This might justify the need for additional objective/ independent assessment obtained from family members or partners in future studies. The third argument might be that this type of scale was not sensitive enough to detect subtle deficits in behavioral functions in HIV-positive subjects, thus explaining the lack of correlations between rating scale and neurometabolite ratios.

When discussing the additional correlations between clinical parameters of HIV infection, sociodemographic variables and BRIEF-A subscales, in HIV-positive group, the significant correlations were observed between Shift scale and age. There were no significant correlations between age and behavioral aspects of EF in HIV-negative group, while there was a significant correlation between age and results on the Shift scale. (The more advanced the age, the worse achievement was detected on this scale). The explanation for the lack of correlations in the HIV-negative group could be the age of the sample (42.18), implying that correlations may be related with advanced age. The pattern of brain aging in HIV-positive population has also been changed with the early introduction of cART into the accelerated aging form (Cole et al., 2017), meaning that HIV infection increases the burden of risk for age-related comorbidities. The presence of the correlation between age and Shift scale, and the absence of any correlations with age in age-matched HIV-negative controls, might be in favor of this assumption.

Key aspects of shifting include the ability to make transitions, tolerate change, solve a problem flexibly, switch or alternate attention, and change focus from one mindset or topic to another. According to the relationship between age and Shift scale, this scale might particularly reflect those aspects of EF that are most vulnerable to the aging effect. This might be important in the context of compliance, working ability and global cognitive efficacy prediction in HIV-positive individuals.

Furthermore, significant correlations were observed between Working memory and current CD4 count (the higher current CD4 count, the worse achievement was detected on this scale) in HIV-positive group; as well as between Working memory and educational achievement (the better educational level, the worse achievement was detected on this scale) in HIV-negative group. These correlations came as a surprise, given that the better educational achievement should be associated with bigger capacity of working memory, while higher current CD4 counts should be associated with better achievements on the Working memory scale. Potential explanations for obtained correlations could be that the current CD4 count–which is correlate of the immune state–does not reflect adequately the protective effect on neuronal function and some aspects of EF, Working memory in the first line. This fact confirms the complex relationship between parameters of HIV infection and EF, stated in recent studies that failed to present correlations between positive indices of immune response (cART compliance, high current CD4 count, high nadir CD4) and improvement in different aspects of cognitive functioning. Indirectly, it can be concluded that there are very complex inter-reactions and correlations between manifest indices of immune response and latent parameters that affect rapid cognitive decline, potentially involved in acceleration of brain aging in HIV-positive individuals.

Potential explanation for unexpected correlations between educational achievement and Working memory scale in control group might be explained in the way that people with higher academic achievements are faced with increased number of cognitive tasks in the modern life settings, they are involved in a daily multitasking thus lowering the capacity of the working memory. Bearing in mind that the working memory is one of our core cognitive functions that allows us to keep information in mind for short period of time and then use this information when needed, representing the capacity to hold information in mind for the purpose of completing a task, encoding information, or generating goals, plans, and sequential steps to achieving goals, it becomes clear that exhausting daily cognitive tasks can create a misbalance in this function.

Also, we observed significant negative correlation between duration of cART and NAA/Cr ratio in the dorsal part of the right ACG, pointing to the decrease of NAA/Cr with increased duration of cART. This introduces the aspect of potential low-level neurotoxicity of the therapy, especially when knowing that most of our patients were on a so-called “old-fashioned” cART. Also, we confirmed the strong correlation between age and NAA/Cr ratios in the dorsal ACG (left and right). This is a known fact, implying that age is a major contributing factor to the decrease of neuronal marker, even greater than HIV-induced neuronal injury (Boban et al., 2017).

Finally, five hierarchical regression analyses were performed in order to determine the influence of the NAA/Cr and Cho/Cr ratios at the dorsal ACG (left and right) on following BRIEF-A scales: Inhibit, Shift, Emotional Control, Self-monitoring, and Plan/Organize. Age was used as a controlling variable in the first step, the current level of CD4 cells was used in the second step, and the ratios of NAA/Cr and Cho/Cr at the dorsal ACG (left and right) in the third step. Age and current CD4 count were chosen as controlling variables since they showed statistically significant correlations with some BRIEF-A subscales. Dependent variables were BRIEF-A subscales, based on statistically significant correlations with neurobiochemical changes. The results (Tables 8–17 in Supplementary Materials) showed that age had a statistically significant influence on clinical scale Shift. However, none of the metabolite ratios had significant influence on BRIEF-A subscales. Potential explanation for the lack of expected influence of neurometabolite ratios on BRIEF-A scales can be sought in the context of significant correlations of neurometabolite ratios among themselves, thus lowering the correlation significance between other unique predictors (Table 5). Only age was a significant predictor of the achievement on Shift scale in HIV-positive subjects, in whom age defines the decrease of the ability to move with ease from one situation, activity, or aspect of a problem to another as the circumstances demand. Even though in our study sample there were no subjects of advanced age, the presence of HIV infection in the middle-aged group has a certain negative effect on behavioral aspects of EF, despite good peripheral control of the disease. In other word, even though the age itself is not expected to affect the cognitive status in the middle-aged group, due to presumed accelerated brain aging in HIV infection, certain impairment might be detected in spite of good disease parameters.

It is important to emphasize that after the introduction of the Bonferroni corrections, none of the initially detected and explained correlations remained significant.

Finally, the authors feel that the lack of correlations after the introduction of the Bonferroni corrections could also be the “victim” of too strict statistical thresholds on the small sample. If the sample was bigger, there is a possibility that initially detected correlations would be still observed after introduction of Bonferroni corrections and stricter significance levels. In the future, studies should be conducted on the bigger samples that include functional and working population of HIV-positive individuals. Additionally, the introduction of traditional measures for EF along with the self-assessment scales could give a more objective and comprehensive insight in correlations between neurometabolite ratios and measurements of EF. Also, the introduction of third party (members of family or partners) as independent raters of EF could be extremely helpful. Considering neuroimaging studies, multi-voxel MRS represents a very useful and sensitive tool for the analysis of neurometabolic profile in an HIV-positive individual. HIV-related neuronal injury is diffuse, and not focal, the use of average values for the left and right hemispheres could be methologically justified in some cases.

Alongside with the limitations of this study, there are some advantages also. This is the first study in HIV-positive individuals that revealed the potential correlations between neurobiochemical profile and assessment scales for EF in adult population. The issue of the use of those correlations in clinical management of these patients was also raised. Finally, this was the first study that used rating scales for the assessment of EF in HIV-positive individuals, thus contributing to the existing knowledge about the role of rating scales in EF in the clinical setting.

## Ethics statement

This study was carried out in accordance with the recommendations of Declaration of Helsinki. The protocol was reviewed and approved by the institutional review board (Ethical Comittee of the Faculty of Medicine, University of Novi Sad) governing each site. All subjects gave written informed consent in accordance with the Declaration of Helsinki.

## Author contributions

All the authors have seen and approved the final version and have contributed significantly to the work with regard to conception and design (VB, SB, and JB), acquisition of the data (VB, JM, JB, DK, and DM), data analysis and interpretation (VB, JM, and JB), as well as drafting the article (VB, JM, and JB) or revising it (SB).

### Conflict of interest statement

The authors declare that the research was conducted in the absence of any commercial or financial relationships that could be construed as a potential conflict of interest.
