# Supplementary material for: Executive Functions Rating Scale and Neurobiochemical Profile in HIV-Positive Individuals
Source: Front Psychol. 2018 Jul 19;9:1238. doi: 10.3389/fpsyg.2018.01238 (PMC6060670; doi:10.3389/fpsyg.2018.01238)
Supplement: Supplementary file 1 [file Table_1.DOC]

Supplementary Material

**Executive functions rating scale and neurobiochemical profile in HIV- positive individuals**

**Vojislava Bugarski Ignjatovic^1^, Jelena Mitrovic^2^, Dusko Kozic^3^, Jasmina Boban^3^, Daniela Maric^4^, Snezana Brkic^4^**

^1^ Faculty of Medicine, Department for Psychology, University of Novi Sad, Novi Sad, Serbia

^2^ Faculty of Philosophy, Department for Psychology, University of Novi Sad, Novi Sad, Serbia

^3^ Faculty of Medicine, Department for Radiology, University of Novi Sad, Novi Sad, Serbia

^4^ Faculty of Medicine, Department for Infectious Diseases, University of Novi Sad, Novi Sad, Serbia

*** Correspondence:**Vojislava Bugarski Ignjatovic, Ph.D.
vojislava.bugarski-ignjatovic@mf.uns.ac.rs

# Supplementary Figures and Tables

**TABLE 8 | Hierarchical regression analysis with dependent variable BRIEF-A scale Inhibit**

| Model | R^2^ | R^2^ Change | F Change | p |
| --- | --- | --- | --- | --- |
| 1 | .000 | .000 | .013 | .909 |
| 2 | .064 | .064 | 2.461 | .125 |
| 3 | .251 | .187 | 1.999 | .118 |

**TABLE 9 | Summary of hierarchical regression analysis in prediction of BRIEF-A scale** Inhibit

| Model | | β | | p | 95.0% Confidence Interval for B | |
| --- | --- | --- | --- | --- | --- | --- |
|  |  |  |  |  | Lower Bound | Upper Bound |
| 1 | Age | .019 | .909 | | -.087 | .098 |
| 2 | Age | -.029 | .861 | | -.100 | .084 |
|  | Current CD4 | .257 | .125 | | -.001 | .005 |
| 3 | Age | -.337 | .091 | | -.203 | .016 |
|  | Current CD4 | .236 | .149 | | -.001 | .005 |
|  | NAA/Cr - Dorsal Part of the ACG right | -.514 | .252 | | -17.131 | 4.654 |
|  | Cho/Cr - Dorsal Part of the ACG right | .093 | .769 | | -11.878 | 15.929 |
|  | NAA/Cr - Dorsal Part of the ACG left | -.036 | .938 | | -11.733 | 10.862 |
|  | Cho/Cr - Dorsal Part of the ACG left | .103 | .779 | | -14.240 | 18.844 |

**TABLE 10 | Hierarchical regression analysis with dependent variable BRIEF-A scale Shift**

| Model | R^2^ | R^2^ Change | F Change | p |
| --- | --- | --- | --- | --- |
| 1 | .153 | .153 | 6.666 | .014 |
| 2 | .174 | .021 | .933 | .341 |
| 3 | .254 | .080 | .860 | .498 |

**TABLE 11 | Summary of hierarchical regression analysis in prediction of BRIEF-A scale Shift**

| Model | | β | p | 95.0% Confidence Interval for B | |
| --- | --- | --- | --- | --- | --- |
|  |  |  |  | Lower Bound | Upper Bound |
| 1 | Age | .391 | .014 | .016 | .134 |
| 2 | Age | .363 | .024 | .010 | .130 |
|  | Current CD4 | .149 | .341 | -.001 | .003 |
|  | Age | .193 | .325 | -.039 | .113 |
|  | Current CD4 | .144 | .372 | -.001 | .003 |
|  | NAA/Cr - Dorsal Part of the ACG right | .165 | .711 | -6.148 | 8.915 |
|  | Cho/Cr - Dorsal Part of the ACG right | -.273 | .388 | -13.740 | 5.487 |
|  | NAA/Cr - Dorsal Part of the ACG left | -.486 | .299 | -11.864 | 3.759 |
|  | Cho/Cr - Dorsal Part of the ACG left | .302 | .410 | -6.747 | 16.128 |

**TABLE 12 | Hierarchical regression analysis with dependent variable BRIEF-A scale Emotional Control**

| Model | R^2^ | R^2^ Change | F Change | p |
| --- | --- | --- | --- | --- |
| 1 | .050 | .050 | 1.954 | .170 |
| 2 | .052 | .002 | .075 | .786 |
| 3 | .209 | .157 | 1.585 | .202 |

**TABLE 13 | Summary of hierarchical regression analysis in prediction of BRIEF-A scale Emotional Control**

| Model | | β | p | 95.0% Confidence Interval for B | |
| --- | --- | --- | --- | --- | --- |
|  |  |  |  | Lower Bound | Upper Bound |
| 1 | Age | .224 | .170 | -.037 | .201 |
| 2 | Age | .216 | .200 | -.044 | .201 |
|  | Current CD4 | .045 | .786 | -.003 | .004 |
| 3 | Age | -.080 | .692 | -.178 | .119 |
|  | Current CD4 | .020 | .905 | -.004 | .004 |
|  | NAA/Cr - Dorsal Part of the ACG right | -.041 | .928 | -15.445 | 14.117 |
|  | Cho/Cr - Dorsal Part of the ACG right | -.073 | .822 | -20.965 | 16.770 |
|  | NAA/Cr - Dorsal Part of the ACG left | -.495 | .304 | -23.202 | 7.461 |
|  | Cho/Cr - Dorsal Part of the ACG left | .307 | .415 | -13.356 | 31.539 |

**TABLE 14 | Hierarchical regression analysis with dependent variable BRIEF-A scale Self-monitoring**

| Model | R^2^ | R^2^ Change | F Change | p |
| --- | --- | --- | --- | --- |
| 1 | .012 | .012 | .467 | .499 |
| 2 | .014 | .001 | .052 | .821 |
| 3 | .226 | .212 | 2.196 | .092 |

**TABLE 15 | Summary of hierarchical regression analysis in prediction of BRIEF-A scale Self-monitoring**

| Model | | β | p | 95.0% Confidence Interval for B | |
| --- | --- | --- | --- | --- | --- |
|  |  |  |  | Lower Bound | Upper Bound |
| 1 | Age | .112 | .499 | -.046 | .093 |
| 2 | Age | .105 | .539 | -.050 | .093 |
|  | Current CD4 | .038 | .821 | -.002 | .002 |
| 3 | Age | -.100 | .617 | -.105 | .063 |
|  | Current CD4 | .051 | .755 | -.002 | .002 |
|  | NAA/Cr - Dorsal Part of the ACG right | .143 | .752 | -7.064 | 9.679 |
|  | Cho/Cr - Dorsal Part of the ACG right | -.514 | .116 | -19.154 | 2.217 |
|  | NAA/Cr - Dorsal Part of the ACG left | -.542 | .256 | -13.612 | 3.753 |
|  | Cho/Cr - Dorsal Part of the ACG left | .371 | .320 | -6.415 | 19.011 |

**TABLE 16 | Hierarchical regression analysis with dependent variable BRIEF-A scale Plan/Organize**

| Model | R^2^ | R^2^ Change | F Change | p |
| --- | --- | --- | --- | --- |
| 1 | .044 | .044 | 1.703 | .200 |
| 2 | .120 | .076 | 3.127 | .085 |
| 3 | .216 | .095 | .974 | .435 |

**TABLE 17 | Summary of hierarchical regression analysis in prediction of BRIEF-A scale Plan/Organize**

| Model | | β | p | 95.0% Confidence Interval for B | |
| --- | --- | --- | --- | --- | --- |
|  |  |  |  | Lower Bound | Upper Bound |
| 1 | Age | .210 | .200 | -.029 | .134 |
| 2 | Age | .157 | .329 | -.041 | .120 |
|  | Current CD4 | .281 | .085 | .000 | .005 |
| 3 | Age | -.011 | .957 | -.104 | .098 |
|  | Current CD4 | .296 | .079 | .000 | .005 |
|  | NAA/Cr - Dorsal Part of the ACG right | -.354 | .439 | -13.928 | 6.189 |
|  | Cho/Cr - Dorsal Part of the ACG right | -.042 | .897 | -13.661 | 12.018 |
|  | NAA/Cr - Dorsal Part of the ACG left | .030 | .950 | -10.111 | 10.755 |
|  | Cho/Cr - Dorsal Part of the ACG left | -.031 | .934 | -15.899 | 14.652 |
